# Irradiation Enhances the Ability of Monocytes as Nanoparticle Carrier for Cancer Therapy

**DOI:** 10.1371/journal.pone.0139043

**Published:** 2015-09-29

**Authors:** Pei-Shin Jiang, Ching-Fang Yu, Chia-Yi Yen, Christopher William Woo, Shao-Hua Lo, Yu-Kuan Huang, Ji-Hong Hong, Chi-Shiun Chiang

**Affiliations:** 1 Department of Biomedical Engineering and Environmental Sciences, National Tsing-Hua University, Hsinchu, Taiwan; 2 Department of Radiation Oncology, Chang Gung Memorial Hospital at Linkou, Taoyuan, Taiwan; 3 Radiation Biology Research Center, Institute for Radiological Research, Chang Gung University / Chang Gung Memorial Hospital, Linkou, Taoyuan, Taiwan; 4 Department of Medical Imaging and Radiological Sciences, Chang Gung University, Taoyuan, Taiwan; Technische Universitaet Muenchen, GERMANY

## Abstract

The tumor-homing ability of monocytes renders them a potential cellular delivery system for alternative cancer therapies, although their migratory ability can be impaired following reagent uptake. Approaches that enhance monocyte tumor homing and promote their migration will improve the clinical value of these cells as cellular carriers. Previous studies have shown that irradiation (IR) can promote macrophage aggregation in hypoxic regions. To investigate whether IR enhances the infiltration of bone marrow-derived monocytes (BMDMs) into tumors, the infiltration of BMDMs from GFP-transgenic mice in a murine prostate adenocarcinoma TRAMP-C1 model was examined by fluorescence microscopy. IR did not increase the number of BMDMs that infiltrated initially, but did increase monocyte retention within IR-treated tumors for up to 2 weeks. We also showed that BMDMs can take up various imaging and therapeutic agents, although the mobility of BMDMs decreased with increasing load. When BMDMs were differentiated in IR-treated tumor-conditioned medium (IR-CM) *in vitro*, the nanoparticle load-mediated inhibition of migration was attenuated. These IR-CM-differentiated BMDMs delivered polymer vesicles encapsulating doxorubicin to radiation therapy (RT)-induced hypoxic tumor regions, and enhanced the efficacy of RT. The prolonged retention of monocytes within irradiated tumor tissues and the ability of IR-CM to enhance the migratory ability of cargo-laden BMDMs suggest that monocytes pre-conditioned by IR-CM can potentially act as cellular carriers for targeted therapy following conventional RT.

## Introduction

The tendency of blood monocytes/macrophages to infiltrate tumors and to accumulate in hypoxic regions [1, 2] has made these cells a potential cellular vehicle for targeting therapeutic agents to tumor sites [3, 4] and therefore prompted research into the use of monocytes or macrophages as delivery vehicles in antiviral [5] or anticancer therapy [3, 4, 6, 7]. The concept of adoptively transferring *ex vivo*-generated macrophages into cancer patients was proposed quite a long time ago. This kind of adoptive transfer was performed for the first time in B16 melanoma model, where infusion of *ex vivo*-activated macrophages suppressed pulmonary metastases [8]. Muthana *et al*. [4] recently demonstrated a promising clinical application for human blood monocyte-derived macrophages, which were able to deliver oncolytic virus to the hypoxic areas of human prostate tumors after the therapy in an orthotopic xenograft model. This indicates that monocytes can be used as cellular carriers to treat hypoxic tumors. Although reports have shown that tumor-associated macrophages (TAMs) are preferentially located at hypoxic regions [2], we have previously shown that the association of TAMs with hypoxia is dependent on the type of tumor and local microenvironmental cues [9, 10]. Approaches that can enhance the trafficking and tissue retention of monocytes within hypoxic regions will increase the clinical value of using monocytes as cellular carriers in cancer therapy.

The recruitment of various blood-borne bone marrow-derived cells (BMDCs) into tumor tissues has been reported in numerous cancer models as a response to inflammatory signals generated by tumor tissues. After infiltrating the tumors, monocytes/macrophages then migrate along defined chemotactic gradients [11], such as gradients of HIF-1 [2, 12], VEGF [13], or SDF-1 [14], to target areas. Treatments that can elicit inflammatory reactions as well as hypoxic signals can be used to enhance the efficacy of monocytes as cellular carriers. Radiation therapy (RT) is a common protocol in cancer therapy and can generate inflammatory responses [15]. Indeed, we have previously shown that radiation-treated, but not anti-angiogenesis agent-treated, tumors [10] or tissues [16] promote the accumulation of CD68^+^ TAMs in avascular hypoxic regions [9]. This indicates that radiation-treated tumors or tissues produce specific factors that attract and retain macrophages in avascular, hypoxic tumor sites.

The ability to engulf various particles renders macrophages superior as carriers compared to other cell types [17–20]; therefore, the use of macrophages as cellular carriers for therapeutic or imaging nanoparticles has been examined in several models [5, 6, 21–24]. In addition to the fact that they are good carriers, the mobility of the cells following pre-loading with reagents is another factor that determines their usefulness in clinic; for instance, the mobility of alveolar macrophages decreases after particle uptake [25]. However, few studies have investigated the changes in macrophage mobility following the uptake of therapeutic drugs. Here, we explored the change in macrophage mobility following nanoparticle uptake, and evaluated the effects of irradiation (IR) on the recruitment and retention of bone marrow-derived monocytes (BMDMs) in a murine prostate tumor model.

## Materials and Methods

### Cells and Animals

The TRAMP-C1 prostate cancer cell line was purchased from the American Tissue Type Collection (ATCC; CRL-2730). Six- to eight-week-old C57BL/6J or C57BL/6-Tg(CAG-EGFP)1Osb/J mice were purchased from the National Laboratory Animal Center, Taiwan. The recommendations of the approved guide for the care and use of laboratory animals by the Institutional Animal Care and Use Committee (IACUC) of National Tsing Hua University, Taiwan (approved number: IACUC:10012), were followed at all times. Tumors were generated by intramuscular inoculation of 3 × 10^6^ viable cells into the thigh. Tumors in the thigh were measured by caliber grossly in three orthogonal axes to determine tumor volumes.

#### Preparation of bone marrow-derived monocytes (BMDMs)

Bone marrow cells were harvested from C57BL/6J or C57BL/6-Tg(CAG-EGFP)1Osb/J mice by flushing the femurs and tibias with 2% fetal bovine serum (FBS) in Roswell Park Memorial Institute (RPMI) medium. Cells were then cultured in differentiation medium (RPMI medium containing 10% FBS, 10 ng/ml recombinant mouse M-CSF (R&D)) for 24 h. The non-adhering cells were collected and cultured in ultralow attachment culture plate (Cat. #: 28009033, Corning). The cells were further differentiated in differentiation medium for 7 days. The differentiation medium was changed every 2 days. Cells were then cultured one more day in condition medium prior to the experiment. The condition medium was made of 50% of differentiation medium and 50% of irradiated or non-irradiated tumor cell medium. Cells were irradiated in log phase by 70 Gy or sham irradiated using a cobalt source with a dose rate of 10 Gy/minute in the Nuclear Science and Technology Development Center, National Tsing Hua University, Taiwan. One day after irradiation, the medium was collected, centrifuged to remove debris, and passed through 2 μm filter.

### Flow cytometry assay

Aliquots of cells (5 x 10^5^) were blocked in 10% normal goat serum in PBS for 15 minutes at room temperature followed by incubation with fluorescent dye-conjugated antibody: FITC-conjugated anti mouse CD11b monoclonal antibodies, PE-conjugated anti mouse CD45 monoclonal antibody, FITC-conjugated anti mouse F4/80 monoclonal antibody, FITC-conjugated anti mouse CD68 cells monoclonal antibody, or PE-conjugated anti mouse CD206 monoclonal antibody for 45 minutes on ice. All antibodies were purchased from BD Biosciences (San Jose, CA, USA). Cells were washed three times in PBS and analyzed by FACSCantoII flow cytometer (BD, Franklin Lakes, NJ, USA)).

### Uptake of nanoparticles by BMDMs

Various nanoparticles such as Dio-labeled perfluropentane droplet prepared by Prof. Chi-Kuang Yeh [26], 3,3-dioctadecyloxacarbocyanine perchlorate (DiO)-laden PAAC-d25 polymer bubbles and doxorubicin (Dox)-loaded PAAC-d15 vesicles (PAAC-Dox) prepared by Prof. Hsin-Chen Chiu [27, 28] were cultured with BMDMs for 4 h. BMDMs pre-loaded with PAAC-Dox were used for *in vitro* study.

### In vitro migration assay

The migration assay was performed as the procedures described in previous publication {Wang, 2012 #250}. Each assay was performed in triplicate and repeated 3 times.

### In vivo tumor model

Tumors were allowed to grow to a size of 5 mm in diameter and then treated by 6-MV X-rays from a linear accelerator at a dose rate of 2–3 Gy/min and a 1.5-cm bolus on the surface. The chemicals or BMDMs (5 x 10^6^ cells/mouse) were intravenously (i.v.) injected at 1, 4, and 7 days after single dose of 25 Gy. The amount of Dox within 5 x 10^6^ cells of BMDMs (ie. BMDMs-PAAC-Dox) was determined by DOX fluorescence intensity at 560 nm of cell lysates and the concentration of Dox of all treatments was adjusted to make sure that all mice were injected with same amount of Dox, which was equivalent to 1 mg Dox/Kg of body weight. Tumor size was measured two ~ three times a week by calipers prior to sacrifice for histology.

### Immunohistochemistry and image analysis

The tissue preparation and staining procedures were the same as described in previous publication {Chen, 2009 #176}. Tumor hypoxia was studied by i.p. injection of 4 mg pimonidazole hydrochloride (Hypoxyprobe™-1 Kit, Hypoxyprobe, Burlington, MA, USA) in 0.1 ml solution 1 h before tumor harvest. Tissues were removed and placed in cold 4% paraformaldehyde overnight then processing and embedding in paraffin or OCT. Ten micrometers cryostat sections were fixed in methanol at −20°C for 10 min, and then rehydrated in PBS. Non-specific binding was blocked by incubating sections in 1% of bovine serum albumin (BSA) in PBS for 30 min. Pimonidazole (PIMO) was detected with mouse antibody (Hypoxyprobe) and goat anti-mouse IgGγ1 Alexa 488 (Invitrogen). To detect tumor hypoxia and its association with vascular endothelial cells, co-staining was performed with a mouse monoclonal antibody (Hypoxyprobe-1 Kit; Chemicon) and a rat anti-mouse CD31 antibody (BD biosciences). To identify macrophages, rat anti-mouse F4/80 (Serotec, Oxford, UK), rat anti-mouse CD68 (Serotec, Oxford, UK), rat anti-mouse CD11b (BD biosciences), and rat anti-mouse CD45 (BD biosciences) were used. Primary antibodies were detected using secondary antibodies labeled with the fluorochromes Alexa Fluor 594 (Molecular Probes), Alexa 488, or anti-rabbit FITC (BD biosciences). To quantitate positive events, each tumor was serially sectioned, 5 sections 100 μm apart were stained, images were captured by AxioCam MRC-5 camera on an Axiovert40 fluorescence microscope (Care Zeiss, Güttingen, Germany) or Laser Scanning Confocal microscope (FluoView 1000, Olympus, Japan), and the total number of positive event was analyzed by Image-Pro 6 (Media Cybernetics) per mouse (n ≧ 3 mice per group).

### Statistical analysis

Statistical analyses were performed using GraphPad Prism 5 software (GraphPad Software, Inc., CA, USA). For all comparisons, statistical significance was tested by one-way ANOVA or Students T test, and *P* < 0.05 was considered statistically significant.

## Results

### Infiltration and differentiation of BMDMs in established tumors

**Fig 1:**
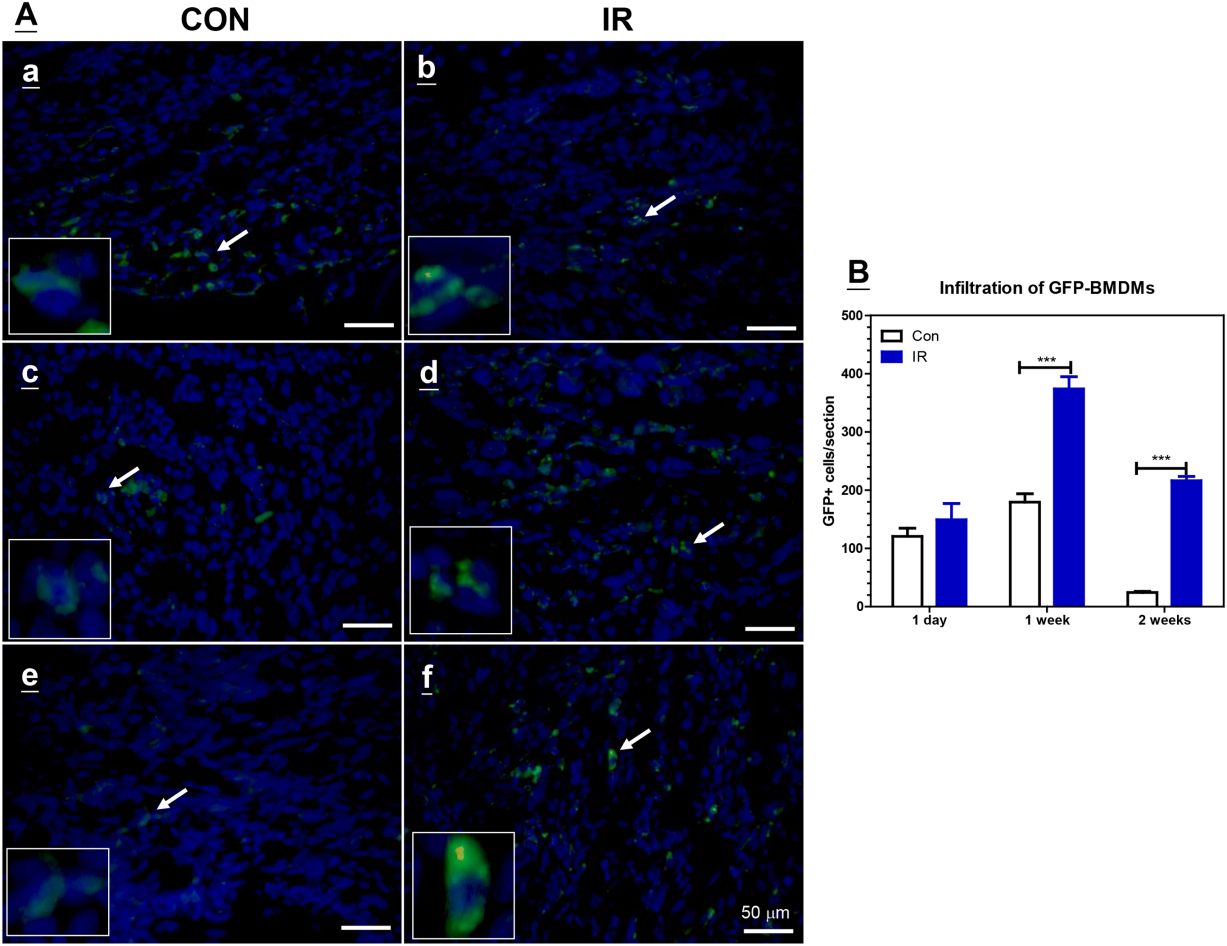
The infiltration of GFP-BMDMs into tumor tissues with (IR) or without (CON) 25 Gy of irradiation. (A) Fluorescence microscopy detection of GFP-BMDMs within brain tumor tissues at 1 day (a and b), one week (c and d), or two weeks (e and f) after the intravenous injection of 2 x 10^6^ GFP-BMDMs. The arrow points to the cells enlarged in the inner square. The scare bar = 50 μm. (B) The quantification of GFP+ cells within tumor tissues. The data represent the average number of GFP+ cells of each section of whole tumor from 3 tumor tissues was counted under 40X objective len. Bar: SE of 3 tumors of each treatment. ***: P< 0.005 by Students T test.

To examine whether BMDMs could act as cellular carriers in cancer radiation therapy, GFP-BMDMs were intravenously (i.v.) injected into C57BL/6J mice with 5-mm diameter TRAMP-C1 tumors in the thigh after a single dose of 25 Gy of radiation. Fluorescence microscopy of the tumor showed that GFP^+^ cells began to appear at tumor edges 1 day after injection (S1 Fig), although there was no significant difference between the control and IR-treated tumors (Fig 1Aa and 1Ab). One week after injection (Fig 1Ac and 1Ad), the number of GFP^+^ cells increased and they were indiscriminately distributed throughout the tumor tissue. While these GFP^+^ cells could still be detected in IR-treated tumors after two weeks (Fig 1A–1f), only a few remained in the control tumors (Fig 1A–1e). Counting of GFP^+^ cells confirmed that IR-treated tumors had more GFP^+^ cells than had the control tumors after both 1 and 2 weeks (Fig 1B).

**Fig 2:**
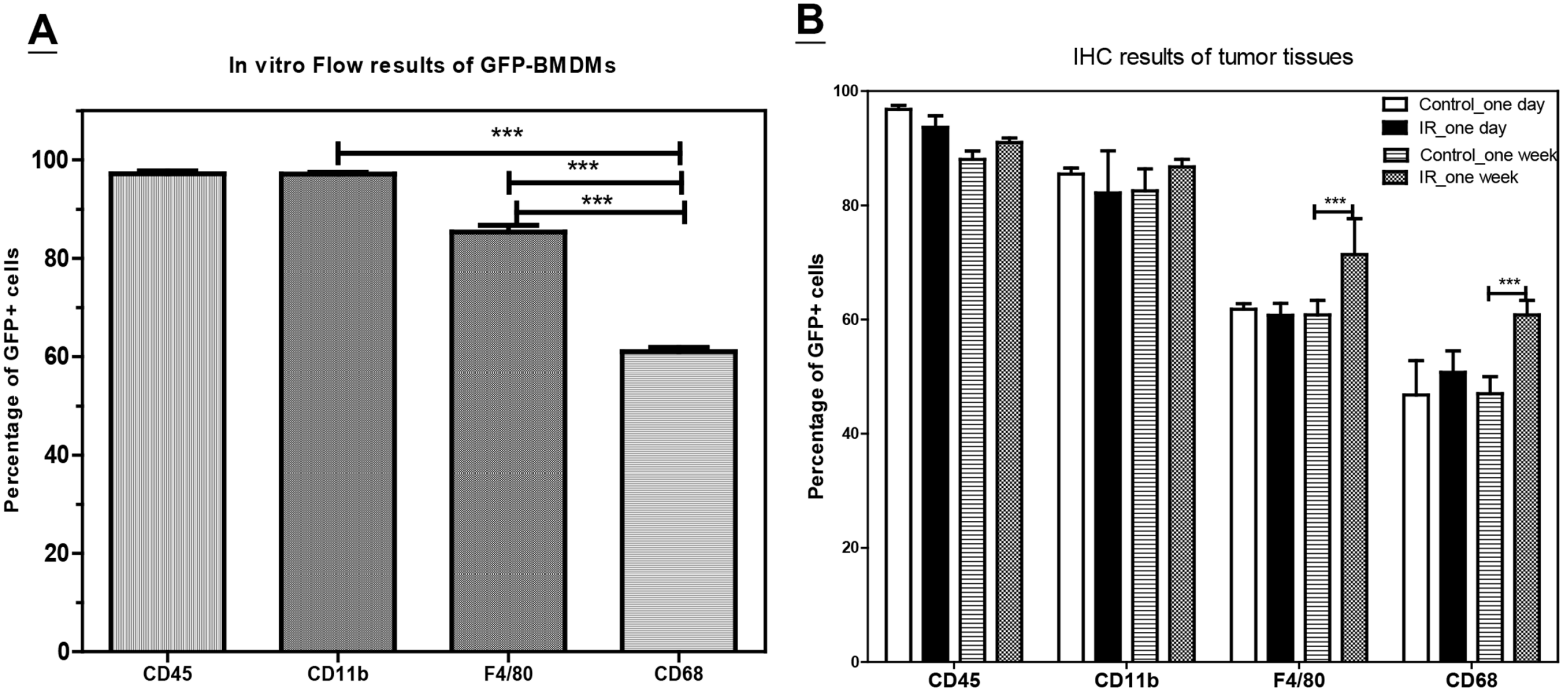
The change of the phenotypes of GFP+ cells within tumors. (A) The percentage of GFP+ cells co-express CD45, CD11b, F4/80, or CD68 antigen as assayed by flow cytometry for the monocytes differentiated from the bone marrow cells harvested from C57BL/6-Tg(CAG-EGFP)1Osb/J mice *in vitro*. Bar: SE of 3 independent experiments. (B) The percentage of GFP+ cells co-express CD45, CD11b, F4/80, or CD68 antigen examined by immunohistochemistry (IHC) within the tumors taken from one day or one week after 25 Gy of irradiation (IR) or shame irradiated (Control). The average percentage of each surface marker of GFP+ cells of 5 randomly selected fields of each tumor from 3 tumor tissues was counted under 40X objective len. Bar: SE of 3 tumor samples. ***: P <0.005 by one way ANOVA test.

To further characterize the phenotype of the infiltrating GFP-BMDMs, flow cytometry (Fig 2) and immunohistochemistry (IHC) (S2 Fig) were used to assess the phenotypic changes of GFP-BMDMs *in vitro* and *in vivo*, respectively. After 8 days of differentiation *in vitro*, all GFP-BMDMs expressed CD45 and CD11b, while 85.4% ± 1.9% expressed F4/80 and 61.0% ± 1.7% expressed CD68^+^ (Fig 2A). Quantification of staining in tumor tissues showed that 97% of GFP^+^ cells in control tumors also expressed CD45, 85% expressed CD11b, 62% expressed F4/80, and approximately 47% were CD68^+^ (Fig 2B). These ratios did not change over time, although these proportions were slightly lower in comparison to the *in vitro* results. IR treatment (25 Gy) did not significantly alter the ratio of CD45^+^ GFP^+^ and CD11b^+^ GFP^+^ cells, although there was a significant increase in the number of CD68^+^ GFP^+^ and F4/80^+^ GFP^+^ cells 1 week after IR (Fig 2B).

### Irradiated TRAMP-C1 tumor-conditioned medium promotes the migration of BMDMs toward tumor-conditioned medium

**Fig 3:**
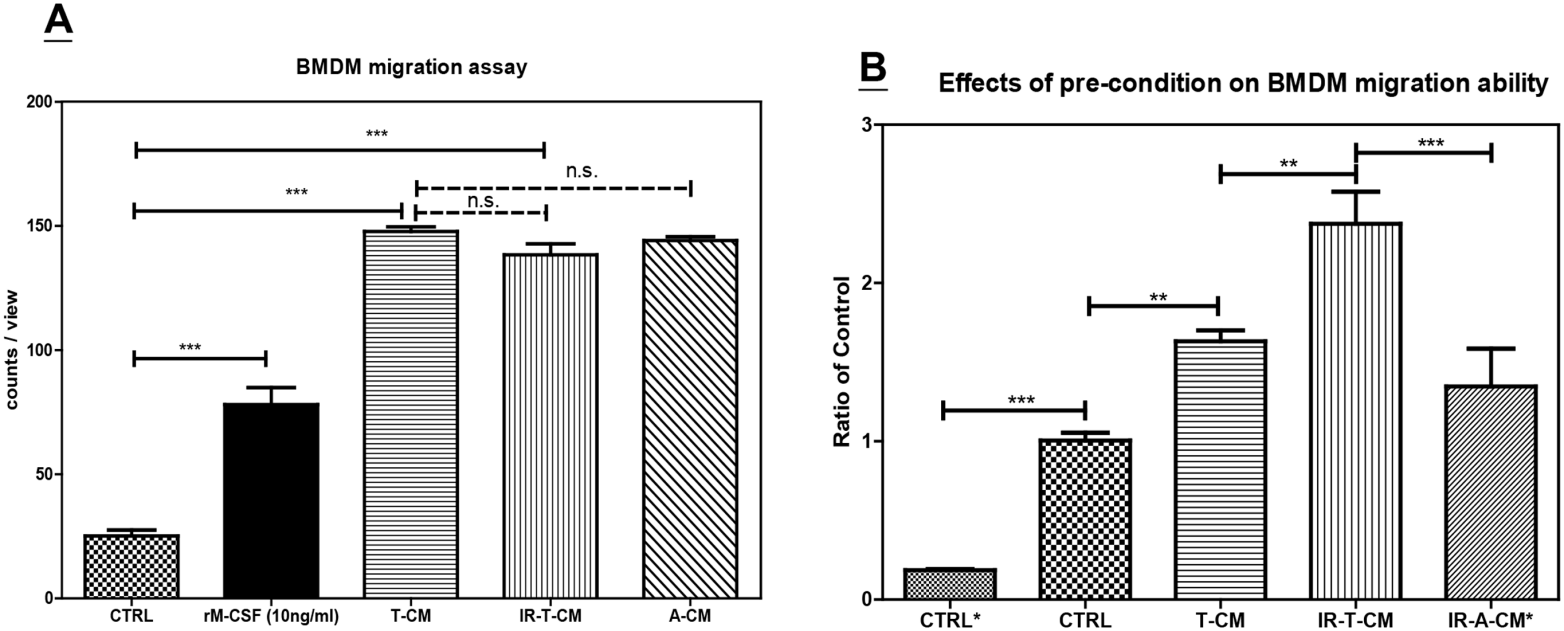
Boyden chamber assay for the migration ability of monocytes. (A) The comparison of the migration ability of monocytes toward different condition medium within the bottom chamber. CTRL: Regular cell culture medium. rM-CSF: Regular cell culture medium containing 10 ng/ml of rM-CSF. T-CM: sham-irradiated TRAMP-C1condition medium. IR-T-CM: 70 Gy-irradiated TRAMP-C1 condition medium. A-CM: sham-irradiated ALTS1C1 condition medium. (B) The influence of pre-condition on BMDM migration ability. CTRL*: BMDMs were pre-conditioned in normal differential serum and then examined for their migration toward normal regular culture medium. CTRL: BMDMs were pre-conditioned in normal differential medium and then examined for their migration toward T-CM. T-CM: BMDMs were pre-conditioned in T-CM for 24 hr and then examined for their migration toward T-CM. IR-T-CM: BMDMs were pre-conditioned in irradiated T-CM for 24 hr and then examined for their migration toward T-CM. IR-A-CM*: BMDMs were pre-conditioned in irradiated T-CM for 24 hr and then examined for their migration toward A-CM. Bar: SE for 8–10 randomly selected fields of each well of 3 independent experiments. **: P<0.01; ***: P <0.005; n.s.: P >0.05 by one way ANOVA test.

To examine if tumor microenvironments produce factors that promote monocyte migration, the migratory ability of BMDMs in various conditioned media was examined via a Boyden chamber assay. TRAMP-C1-conditioned medium (T-CM) was more effective than normal culture medium (CTRL) or medium containing 10 ng/ml rM-CSF at eliciting BMDM migration (Fig 3A). However, the rate of migration of BMDMs toward irradiated TRAMP-C1-conditioned medium (IR-CM) or another medium conditioned by murine astrocytoma cell line ALTS1C1 (A-CM) was similar to that at which these cells migrated toward T-CM. This indicates that tumor microenvironments express factors that attract monocytes, and explains why GFP-BMDMs have a similar initial infiltration rate into the control and irradiated-TRAMP-C1 tumors. However, these data cannot explain why irradiated tumors contain more GFP-BMDMs at 1 and 2 weeks after RT. To further examine if irradiated tissues affect BMDM migratory ability, BMDMs were pre-conditioned in different conditioned media for 1 day prior to the migration assay. We found that BMDMs pre-conditioned in T-CM demonstrated increased migratory ability toward T-CM than regular differentiation medium (CTRL), and this ability was further enhanced if BMDMs were first cultured in IR-T-CM (Fig 3B). However, both pre-conditions did not affect the migration of BMDMs toward normal culture medium (S3 Fig). Furthermore, this effect was tumor-specific, as cells migrated faster towards TRAMP-C1-conditioned medium than towards ALTS1C1-conditioned medium (IR-A-CM* in Fig 3B). Together, these results indicate that, after entering irradiated tumor microenvironments, macrophages may gain increased ability to migrate within tumors.

**Fig 4:**
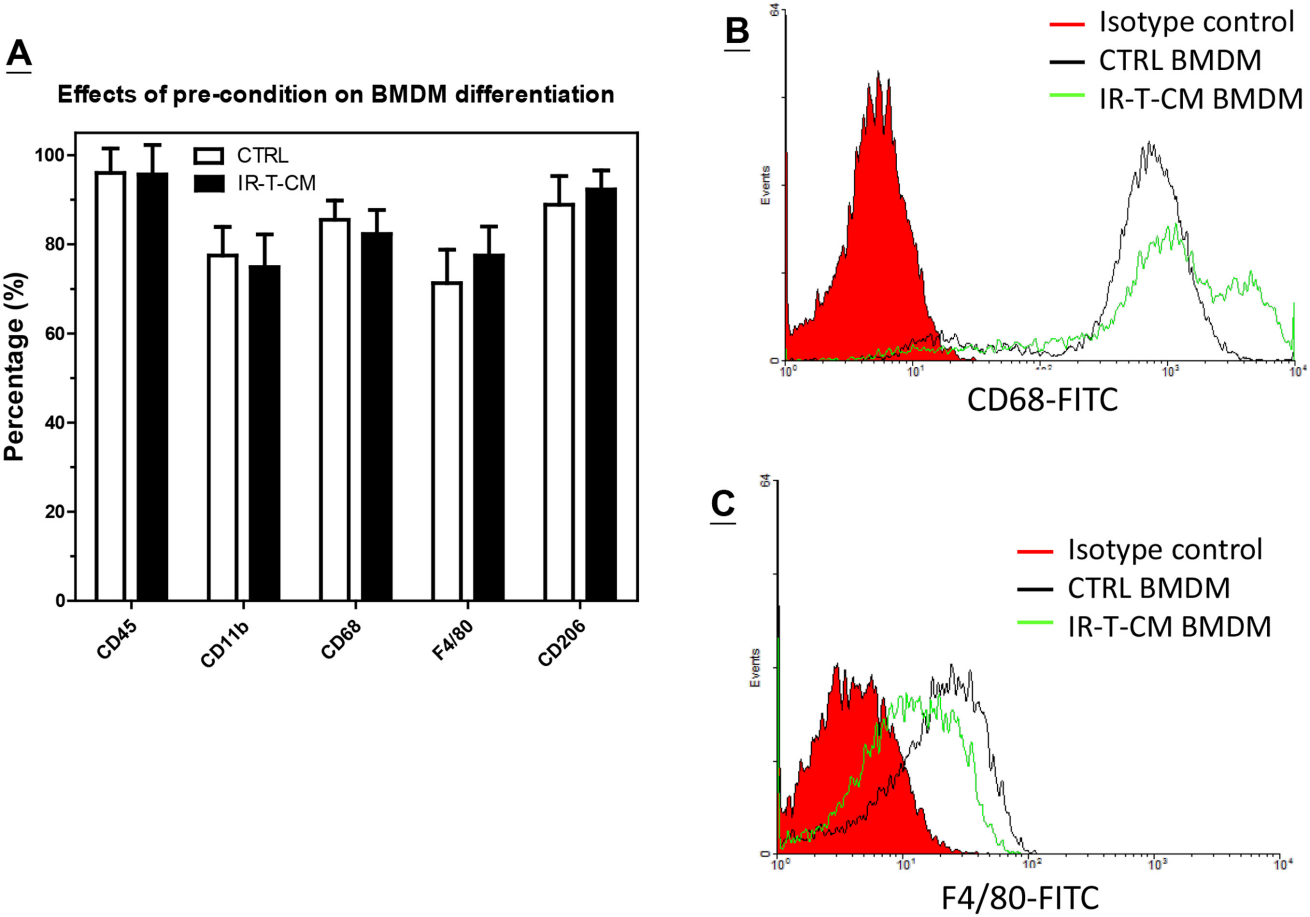
Effect of pre-condition on BMDM phenotype. (A) The percentage of BMDM cells containing CD45, CD11b, CD68, F4/80, or CD206 surface antigens was examined by flow cytometry. CTRL: Bone marrow-derived cells were differentiated in regular BMDM differentiation medium for 8 days. IR-T-CM: Bone marrow-derived cells were differentiated in regular BMDM differentiation medium for 7 days and further cultured in irradiated TRAMP-C1 condition medium (IR-T-CM) for 24 hrs. (B) The histogram of the intensity of FITC-conjugated anti-CD68 antibody for BMDM differentiated from different condition medium. (C) The histogram of the intensity of FITC-conjugated anti-F4/80 antibody for BMDM differentiated from different condition medium. Bar: SE of 3 independent experiments.

To further explore the effects of pre-conditioning on the migratory ability of macrophages, flow cytometry was used to examine the expression of macrophage-associated differentiation markers CD45, CD11b, CD68, F4/80, and CD206 to characterize macrophage phenotypes. The percentage of each surface marker did not differ significantly between the control BMDMs and those pre-conditioned in IR-T-CM (Fig 4A). Further, flow cytometric histograms show that the mean fluorescence intensity (MFI) of these surface markers in control BMDMs versus IR-CM BMDMs also did not differ significantly (S4 Fig), with the exception of CD68 (Fig 4B) and F4/80 (Fig 4C). The single CD68 peak (MFI: 770) in control BMDMs increased and split into two peaks, namely, CD68^mid^ and CD68^high^ peaks, with MFIs of 787 and 4337, respectively (Fig 4B). The MFI of F4/80 decreased from 9.7 to 5.7. Although we have not been able to identify the major factor contributing to this difference, these results indicate that one-day culture in RT-CM during differentiation can alter the phenotype and migratory characteristics of BMDMs.

### Pre-conditioned BMDMs recovered from uptake-induced migration loss

**Fig 5:**
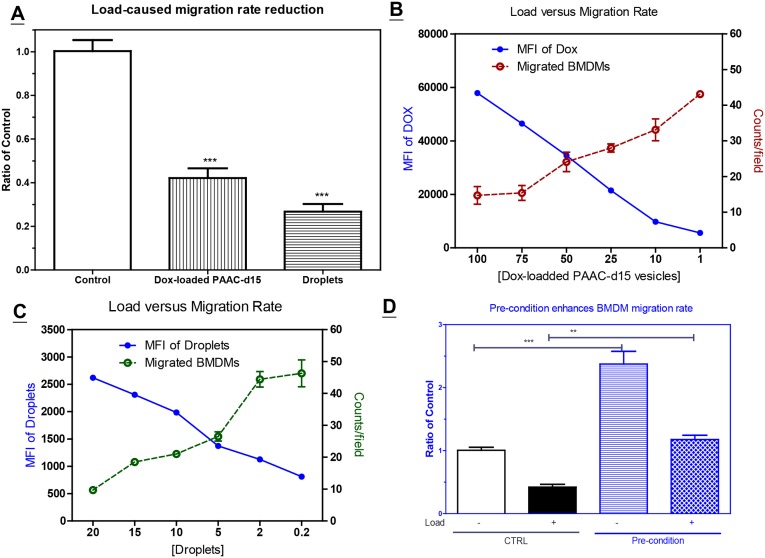
The migration rate of BMDM is reduced after taking up the particles. (A) The migration rate of BMDM is decreased after uptaking up the Dox-loaded PAAC-d15 vesicles or Dio-lableled perfluoropentane droplets. (B) A titration assay for the inverse relationship between the amount of Dox-loaded PAAC-d15 vesicles (as shown by the MFI of Dox on left axis measured by flow cytometry) and the number of BMDM migrated through Boyden chamber (as shown by the counts/field on right axis). (C) A titration assay for the inverse relationship between the amount Dio-labeled perfluoropentane droplets (as shown by the MFI of DiO on left axis) and the number of BMDM migrated through Boyden chamber (as shown by the counts/field on right axis). (D) Comparison of the migration rate of BMDM pre-cultured in regular (CTRL) versus IR-T-CM (Pre-condition) for 24 hrs. Bar: SE for 8–10 randomly selected fields of each well of 3 independent experiments. ***: P <0.005; **: P<0.01 by one way ANOVA test.

Many studies have shown that monocytes and macrophages have a great capacity for taking up various nanoparticles [17–20]. In this study, BMDMs were able to take up nanoparticles (S5 Fig) such as DiO-laden PAAC-d25 polymer bubbles, Dox-loaded PAAC-d15 vesicles, and DiO-labeled perfluoropentane droplets. In experimental models with Dox-loaded PAAC-d15 vesicles and DiO-labeled perfluoropentane droplets, BMDM mobility decreased after taking up the particles (Fig 5A), as shown via migration assays, and a titration assay showed that BMDM mobility negatively correlated with the quantity of pre-loaded particles (Fig 5B and 5C). While these data confirm that BMDMs can be cellular carriers for imaging or therapeutic nanoparticles, they also indicate that BMDM migration is impaired after cargo uptake. However, the payload-induced migration loss was mitigated in IR-T-CM-derived BMDMs, although these cells were still slower than un-loaded, pre-conditioned BMDMs (Fig 5D).

### BMDM delivery of therapeutic nanoparticles enhances the efficacy of radiation therap*y*

**Fig 6:**
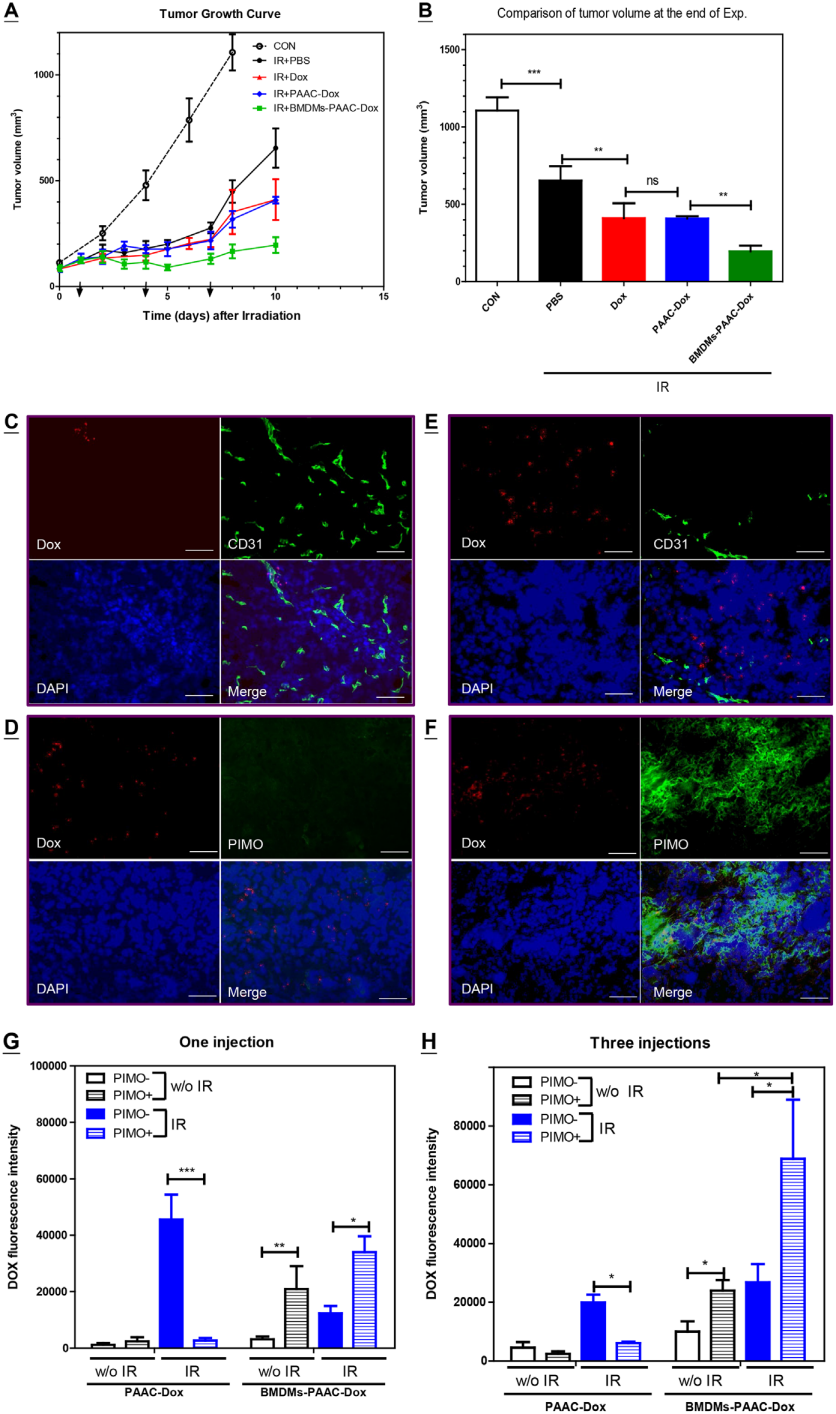
BMDM delivery of therapeutic nanoparticles enhances the efficacy of radiation therapy. (A) Tumor growth curve of TRAMP-C1 tumors growing subcutaneously in the thigh following various combination treatments. IR: 25 Gy of radiation was given when tumor diameter is around 5 mm. PBS, Dox, PAAC-Dox or BMDMs-PAAC-Dox was given intravenously at 1, 4, and 7 days after radiation treatment. The data represent the average of three to five mice from each group of one representative data set of 3 independent repeated experiments. (B) Comparison of tumor volume at the end of experiments. Bar: SE. **: P<0.01; ***: P <0.005; n.s.: P >0.05 by one way ANOVA test. (C) and (D) Representative fluorescent imaging of tumor tissues obtained from one day after the last administration of PAAC-Dox. (E) and (F) Representative fluorescent imaging of tumor tissues obtained from one day after the last administration of BMDM-PAAC-Dox. scale bar = 50 um. Comparison of DOX fluorescent intensity within PIMO negative versus PIMO positive region after one injection (G) or three injections (H) of PAAC-Dox versus BMDMs-PAAC-Dox between tumor with (IR) and without (w/o IR) 25 Gy of irradiation. The data represent the average Dox fluorescent intensity within PIMO- or PIMO+ region of 5 randomly selected fields of each tumor from 3 tumor tissues was analyzed by Image Pro 6 under same exposure time of 40X objective lens imaging. Bar: SE. ***: P <0.005; **: P<0.01; *: P<0.05 by one way ANOVA test.

To determine if pre-conditioned BMDMs can carry drug-loaded nanoparticles as an adjuvant for radiotherapy, doxorubicin (Dox) was first encapsulated in PAAC-d15 vesicles, which can prevent Dox-induced cytotoxicity in macrophages for up to 72 h *in vitro* (data not shown). BMDMs were first pre-cultured in IR-T-CM for 1 day, then with Dox-encapsulated PAAC-d15 vesicles (PAAC-Dox) for 4 h prior to injection. The concentration of PAAC-Dox vesicles was determined by a titration curve (Fig 5) to optimize the Dox load against attenuation of migration rate. The concentration of Dox within BMDMs was quantified spectrometrically, and was used to evaluate the quantity of Dox and PAAC-Dox administered (equivalent to 1 mg Dox/kg of body weight). Free Dox, PAAC-Dox, BMDMs, or BMDMs bearing PAAC-Dox (BMDMs-PAAC-Dox) were intravenously injected into mice bearing 5-mm diameter TRAMP-C1 tumors after 1, 4, and 7 days of a single dose of 25 Gy radiation therapy. The tumor growth curve shows that free Dox, PAAC-Dox, and BMDMs-PAAC-Dox alone (Figure A and B in S6 Fig), but not PAAC, BMDMs, or BMDMs-PAAC (data not shown), reduced tumor growth. A single dose of 25 Gy delivered to a tumor approximately 5 mm in diameter (IR) delayed tumor growth for about 4 days (Fig 6A). This effect of IR was further enhanced when Dox, PAAC-Dox, or BMDMs-PAAC-Dox were administered after IR. Comparison of the tumor volumes following each treatment at the end of experimentation (Fig 6B) showed that the adjuvants, free Dox, PAAC-Dox, and BMDMs-PAAC-Dox, significantly enhanced the IR-induced tumor growth delay. Maximal tumor growth suppression was achieved following IR with the administration of BMDMs-PAAC-Dox.

To further examine the spatial distribution of Dox delivered under different protocols, tumor tissues 1 day after each injection were subjected to immunohistochemical staining (IHC). PAAC-d15 vesicle-encapsulated Dox delivered by BMDMs co-localized with CD11b^+^ macrophages on day 1 after the first injection (Figure C in S6 Fig), confirming that PAAC-d15 vesicles can prevent Dox-mediated cytotoxicity in the carrier for up to 72 h. IHC further showed that Dox with or without polymeric vesicle encapsulation, delivered by circulation, was primarily distributed within a 100-μm range of CD31^+^ vessels (Fig 6C) and in PIMO negative regions (Fig 6D). When Dox was encapsulated within PAAC-d15 vesicles and carried by BMDMs, however, the Dox signal could be found as far as 100 μm away from the vessels (Fig 6E) and within the PIMO^+^ hypoxic region (Fig 6F). To confirm if the enhanced effect of IR and BMDMs-PAAC-Dox was associated with the hypoxic tropism of BMDMs, the intensity of the fluorescent Dox signal in PIMO^+^ and PIMO^−^ regions 1 day after the first or last injection (Fig 6G and 6H) was compared in the treatment groups with and without radiation pre-exposure. We found that the Dox delivered by polymeric vesicles was homogeneously distributed within single-treatment tumors (i.e., without IR). No significant difference was observed between PIMO^+^ and PIMO^−^ regions in single-treatment tumors after either 1 or 3 injections. On the other hand, the Dox fluorescence intensity in single-treatment tumors delivered by BMDMs-PAAC was higher in PIMO^+^ regions regardless of the time point after treatment. Pre-exposure of tumors to IR increased the distribution of Dox delivered by PAAC-d15 vesicles to the PIMO^−^ regions 1 day after the first injection, but had less effect after 3 injections. In contrast, pre-exposure of tumors to RT did not affect Dox accumulation in PIMO^+^ regions for tissues obtained 1 day after the first injection, although the relative Dox intensity in PIMO^+^ regions was further enhanced after 3 injections.

## Discussion

The ability of macrophages to engulf foreign particles and infiltrate tumors has prompted researchers to investigate macrophages as potential cellular carriers for therapeutic or imaging agents. Although a recent report by Choi *et al*. [21] confirmed the therapeutic potential of LP-Dox-loaded peritoneal macrophages in delaying tumor growth, the effect was still very limited. In the current study, we demonstrate that BMDMs pre-cultured in irradiated tumor cell-conditioned medium can enhance the delivery of a therapeutic drug to hypoxic regions, and that these cells can be an effective adjuvant for radiation therapy in a murine prostate tumor model.

The initial *in vivo* experiment using GFP-tagged BMDMs demonstrated that GFP-BMDMs were able to infiltrate growing tumors, although they were mainly situated at the tumor edge, i.e., areas with greater perfusion [29]. The number of infiltrating GFP-BMDMs in control tumors remained the same at week 1, but was decreased at week 2 post-injection. This may explain why weekly injections of BMDMs over a period of 5 weeks were required to observe significant growth delay in previous study [21].

In the present study, IR did not affect initial BMDM infiltration, but irradiated tumors were able to consistently attract BMDMs for a longer period of time and into deeper tissues, than were control tumors, for up to approximately 1–2 weeks. This could be one advantage of combining BMDM-mediated drug delivery with RT. Our therapeutic model did demonstrate that the efficacy of RT was increased following 3 injections of PAAC-Dox-loaded BMDMs. Comparison of Dox density in PIMO^+^ versus PIMO^−^ regions clearly shows that pre-conditioned BMDMs can deliver drugs to hypoxic areas more effectively than to non-hypoxic regions (Fig 6). Of interest, the last injection was less effective than the first injection (day 2 versus day 8 of Fig 6G and 6H, respectively), and IR significantly reduced Dox accumulation in tissues when the drug was not delivered by BMDMs. This is likely associated with the IR-mediated reduction in microvessel density [10]. In contrast, IR did not influence Dox accumulation when it was delivered by BMDMs, and the last injection was more effective than the first. These data indicate that irradiated tissues express factors that promote BMDM infiltration, as well as factors that can enhance the targeting of these cells to hypoxic tumor regions. Therefore, these results demonstrate that pre-conditioned BMDMs can act as effective adjuvant drug carriers following RT.

Our *in vitro* experiment confirms that BMDMs are excellent cellular carriers for various imaging molecules or nanoparticles, but their migratory ability decreased proportionally with increasing load. It is therefore necessary to compromise on the load to mitigate the decreased migration rate prior to using BMDMs as cellular carriers for nanoparticles. While early studies have shown that IR can increase the number of TAMs, we were surprised to find that the initial infiltration of BMDMs was similar in control and irradiated tumors. The *in vitro* migration assay demonstrated that BMDMs migrate faster towards TRAMP-C1 CM, although the migration rates toward irradiated TRAMP-C1 CM or ALTS1C1 CM were similar. This partially explains the similar initial infiltration of BMDMs between control and irradiated tumors (Fig 1B), although we cannot conclude if irradiated TRAMP-C1 tissues have the same chemoattractant effect as the control tumor.

Moreover, we found that the migration rate of BMDMs increased when differentiated BMDMs were pre-cultured for 1 day in tumor-conditioned medium. This increased migration rate could compensate for the loss in migration rate due to the payload. Furthermore, the irradiated TRAMP-C1 CM was more effective than control TRAMP-C1 CM in pre-conditioning BMDMs for migration towards TRAMP-C1 CM; this effect was tumor-specific as they migrated more quickly towards TRAMP-C1 CM than ALTS1C1 CM, with no effect on migration towards normal serum. These results establish a new protocol for using pre-conditioned BMDMs as cellular carriers for imaging or therapeutic nanoparticles.

Using the proposed protocol, we successfully demonstrated that pre-conditioned BMDMs efficiently deliver Dox-loaded PAAC-d15 vesicles to irradiated tumors, enhancing the efficacy of radiation therapy, suggesting potential for use in clinical treatment of tumors. More interestingly, IHC showed that the majority of Dox-PAAC particles delivered by BMDMs were located far from the vessels, in contradistinction to those delivered by sole i.v. injection, which are principally localized in areas surrounding vessels. This finding is in agreement with the general concept that the mechanism of nanoparticle-mediated drug delivery is mainly via an enhanced permeability and retention (EPR) effect.

BMDMs can deliver Dox-PAAC more deeply into the tissue, including hypoxic regions, which are relatively inaccessible by circulation and diffusion. Tumor cells within hypoxic regions have been considered as main obstacle in cancer therapy. Moreover, recurrent tumors frequently have higher ratios of hypoxic tumor cells, which may partially explain the refractory responses of these tumors to second treatments. The ability of BMDMs to deliver PAAC-Dox into PIMO^+^ hypoxic regions renders them a potential cellular drug carrier for recurrent tumors. They are particularly useful for IR-induced recurrent tumors because these types of tumors not only contain a higher ratio of hypoxic tumor cells [10], but they also promote macrophage accumulation in hypoxic areas [9, 10]. The higher Dox density in PIMO^+^ regions of IR-treated tumors supports the notion that BMDMs would be particularly suited to treating IR-induced recurrent tumors.

In summary, this study demonstrates that pre-treatment of BMDMs by irradiated tumor-conditioned medium can enhance the potential of BMDMs to function as cellular carriers for therapeutic agents following radiation therapy.

## Supporting Information

S1 Fig The infiltration of GFP-BMDMs into tumor tissues. The infiltration of GFP-BMDMs into tumor tissues. Tumor tissues were collected one day after 25 Gy of irradiation (IR) following by the intravenous injection of GFP-BMDMs into the mice bearing 5 mm in diameter of TRAMP-C1. Tumor border is marked by solid white line. The dot white regions indicate with the infiltration of GFP-BMDMs were 10x Scale bar: 100mm; 40x Scale bar: 20mm.
(TIF)

S2 Fig Confocal imaging of GFP-BMDMs in tumor tissues. The confocal microscopy to examine the differentiation marker of GFP-BMDMs in tumor tissues. Representing pictures to show the co-expression of CD11b or CD68 differentiation marker by GFP-BMDMs. Pictures were taken under 100x objective len. Scale bar = 10mm.
(TIF)

S3 Fig Pre-condition has not effect on BMDM migration ability toward normal culture medium. Pre-condition has no effect on BMDM migration ability toward regular culture medium. All assays were performed in the bottom chamber containing regular culture medium. CTRL: BMDMs were pre-conditioned in regular differentiation medium. T-CM: BMDMs were pre-conditioned for 24 hr in T-CM. IR-T-CM: BMDMs were pre-conditioned for 24 hr IR-T-CM.
(TIF)

S4 Fig Effects of pre-condition on macrophage phenotype. The histogram of the fluorescent intensity of PE-conjugated anti-CD45, FITC-conjugated anti-CD11b or PE-conjugated anti CD206 antibody for BMDM differentiated from different condition medium.
(JPG)

S5 Fig The uptake of BMDM for various nanoparticles. The uptake of BMDM for various nanoparticles. (A) The fluorescent microscopy of DIO-CHF-PAAC-d25 vesicles uptaken by BMDMs. The inner figure is the histogram of flow cytometry result for DIO fluorescence. (B) The fluorescent microscopy of Dox-loaded PAAC-d15 vesicles vesicles uptaken by BMDMs. The inner figure is the histogram of flow cytometry result for Dox fluorescence. (C) The fluorescent microscopy of Perfluropentance droplet uptaken by BMDMs. The inner figure is the histogram of flow cytometry result for DIO fluorescence.
(TIF)

S6 Fig Tumor growth after treatment. (A) Tumor growth curve of TRAMP-C1 tumors growing subcutaneously in the thigh following various single treatment. RT: 25 Gy of radiation was given when tumor diameter is around 5 mm. PBS, Dox, PAAC-Dox or BMDMs-PAAC-Dox was given intravenously at 1, 4, and 7 days after sham radiation treatment. (B) Comparison of tumor volume at the end of experiments. **: P<0.01; ***: P <0.005; n.s.: P >0.05 by one way ANOVA test. ***: P <0.005; *: P<0.05 by one way ANOVA test. (C) Representative fluorescent imaging of tumor tissues obtained from one day after the first administration of BMDMs-PAAC-Dox. scale bar = 50 um.
(TIF)
