# Supplementary figures and images for: Irradiation Enhances the Ability of Monocytes as Nanoparticle Carrier for Cancer Therapy
Source: PLoS One. 2015 Sep 29;10(9):e0139043. doi: 10.1371/journal.pone.0139043 (PMC4587928; doi:10.1371/journal.pone.0139043)

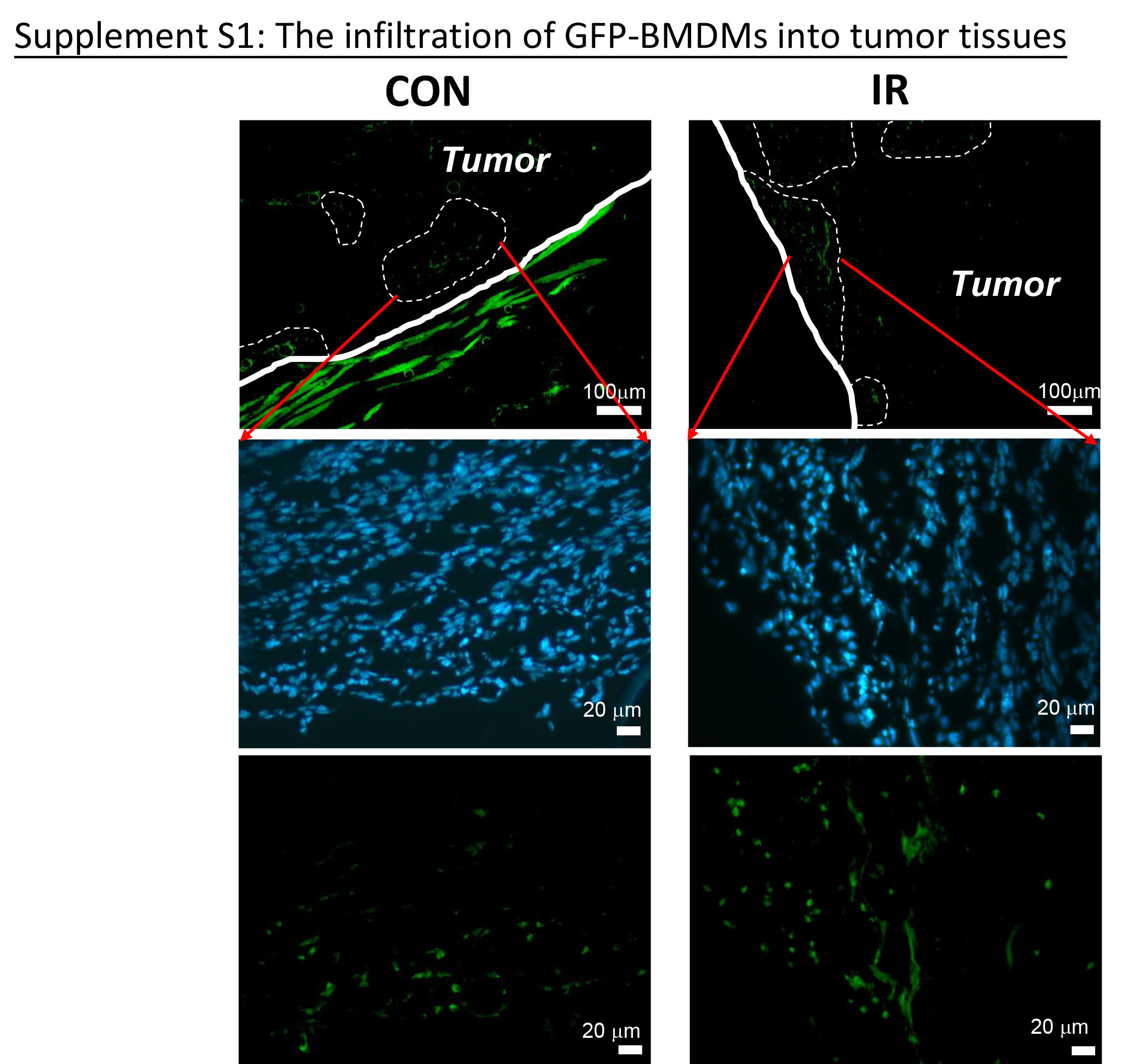

Supplement: S1 Fig — The infiltration of GFP-BMDMs into tumor tissues. Tumor tissues were collected one day after 25 Gy of irradiation (IR) following by the intravenous injection of GFP-BMDMs into the mice bearing 5 mm in diameter of TRAMP-C1. Tumor border is marked by solid white line. The dot white regions indicate with the infiltration of GFP-BMDMs were 10x Scale bar: 100mm; 40x Scale bar: 20mm. (TIF) [file pone.0139043.s001.tif]

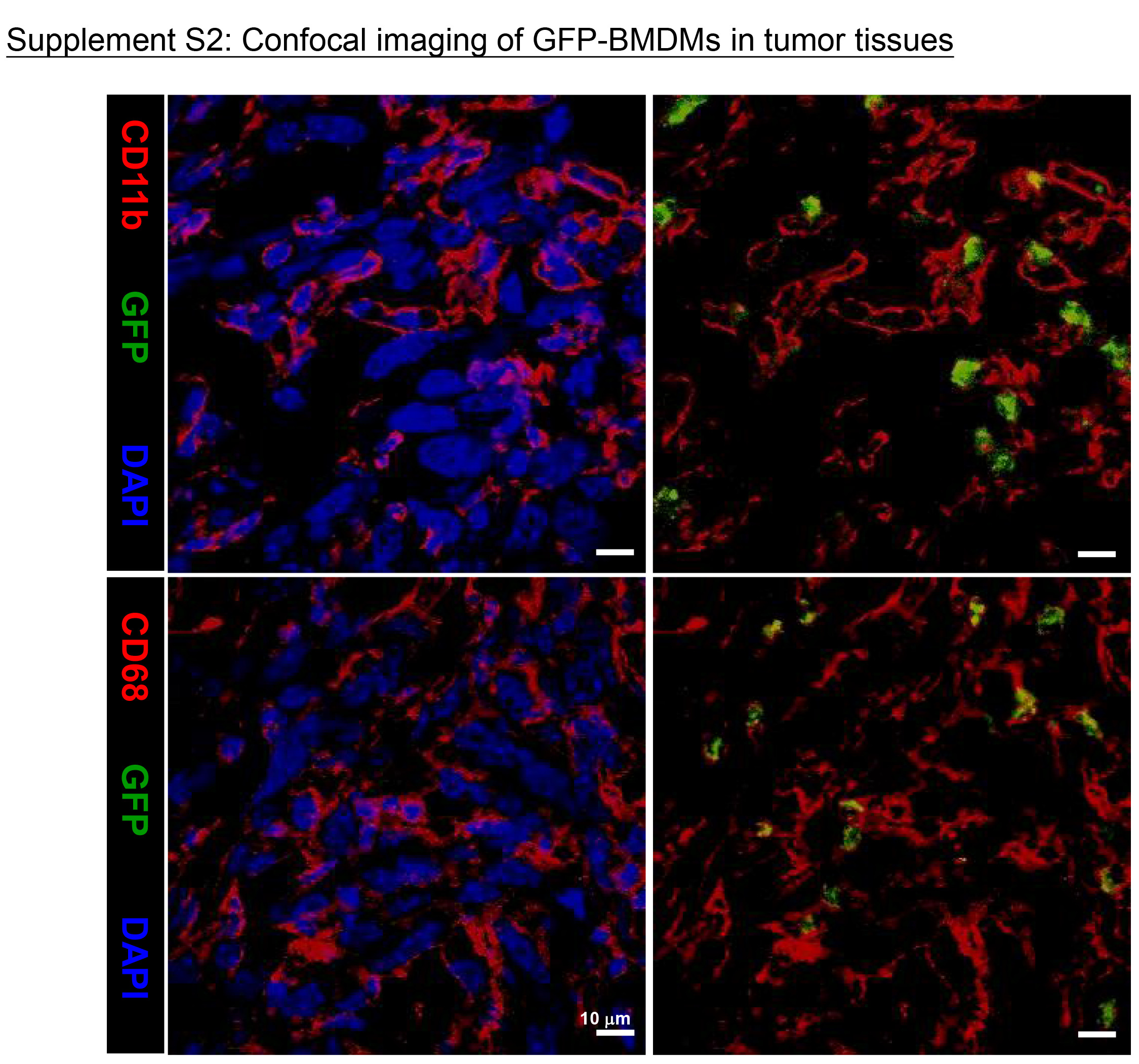

Supplement: S2 Fig — The confocal microscopy to examine the differentiation marker of GFP-BMDMs in tumor tissues. Representing pictures to show the co-expression of CD11b or CD68 differentiation marker by GFP-BMDMs. Pictures were taken under 100x objective len. Scale bar = 10mm. (TIF) [file pone.0139043.s002.tif]

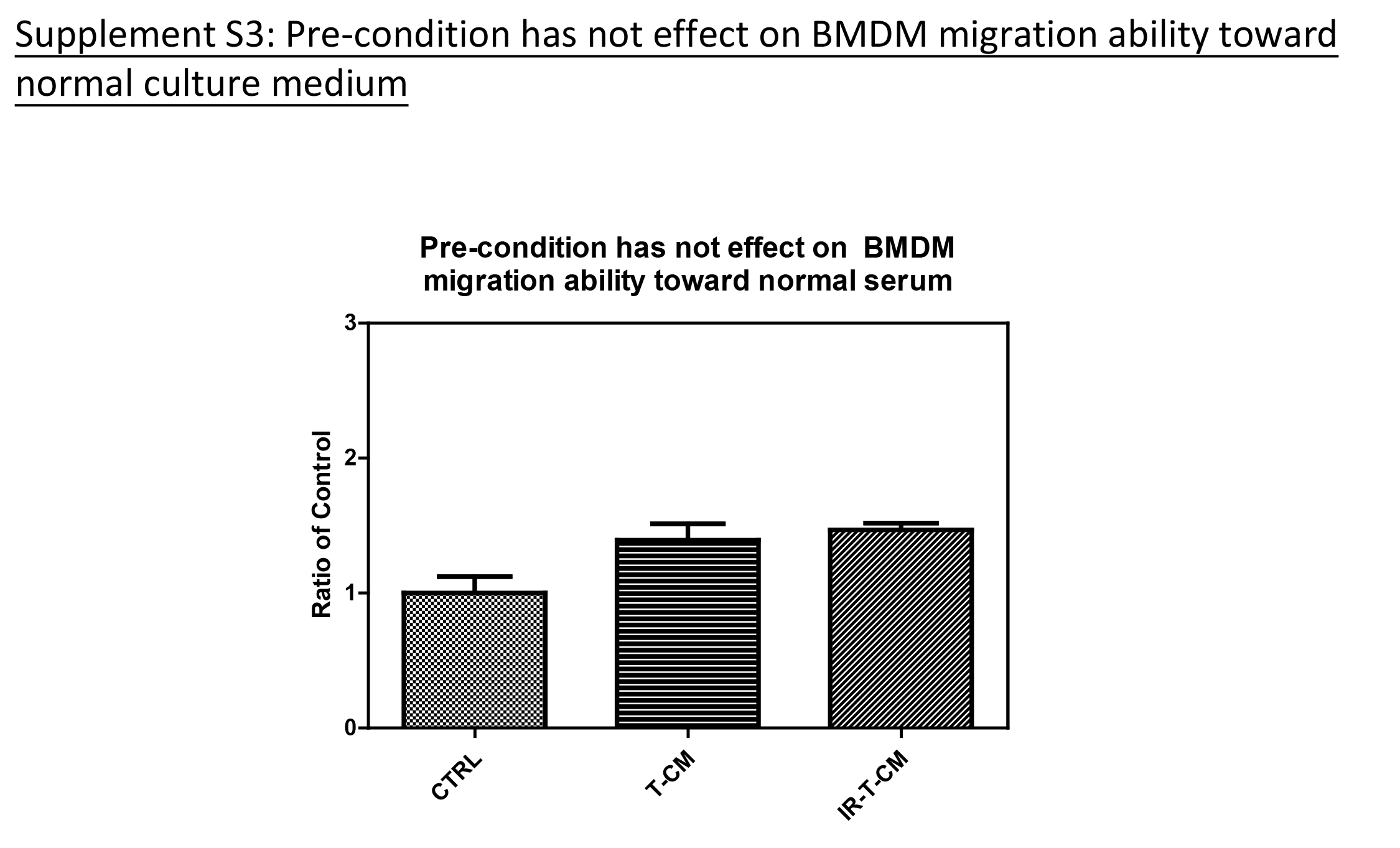

Supplement: S3 Fig — Pre-condition has no effect on BMDM migration ability toward regular culture medium. All assays were performed in the bottom chamber containing regular culture medium. CTRL: BMDMs were pre-conditioned in regular differentiation medium. T-CM: BMDMs were pre-conditioned for 24 hr in T-CM. IR-T-CM: BMDMs were pre-conditioned for 24 hr IR-T-CM. (TIF) [file pone.0139043.s003.tif]

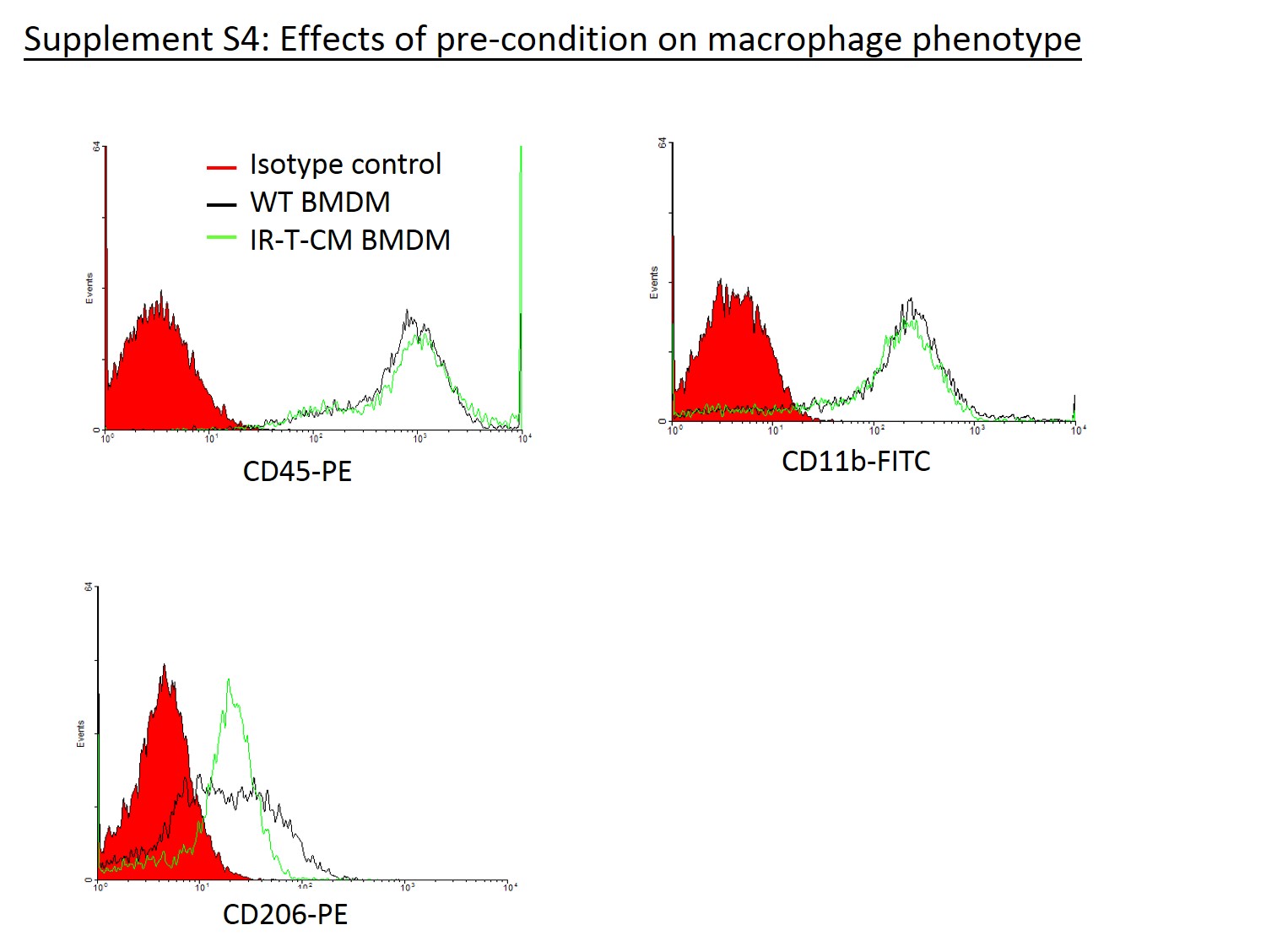

Supplement: S4 Fig — The histogram of the fluorescent intensity of PE-conjugated anti-CD45, FITC-conjugated anti-CD11b or PE-conjugated anti CD206 antibody for BMDM differentiated from different condition medium. (JPG) [file pone.0139043.s004.jpg]

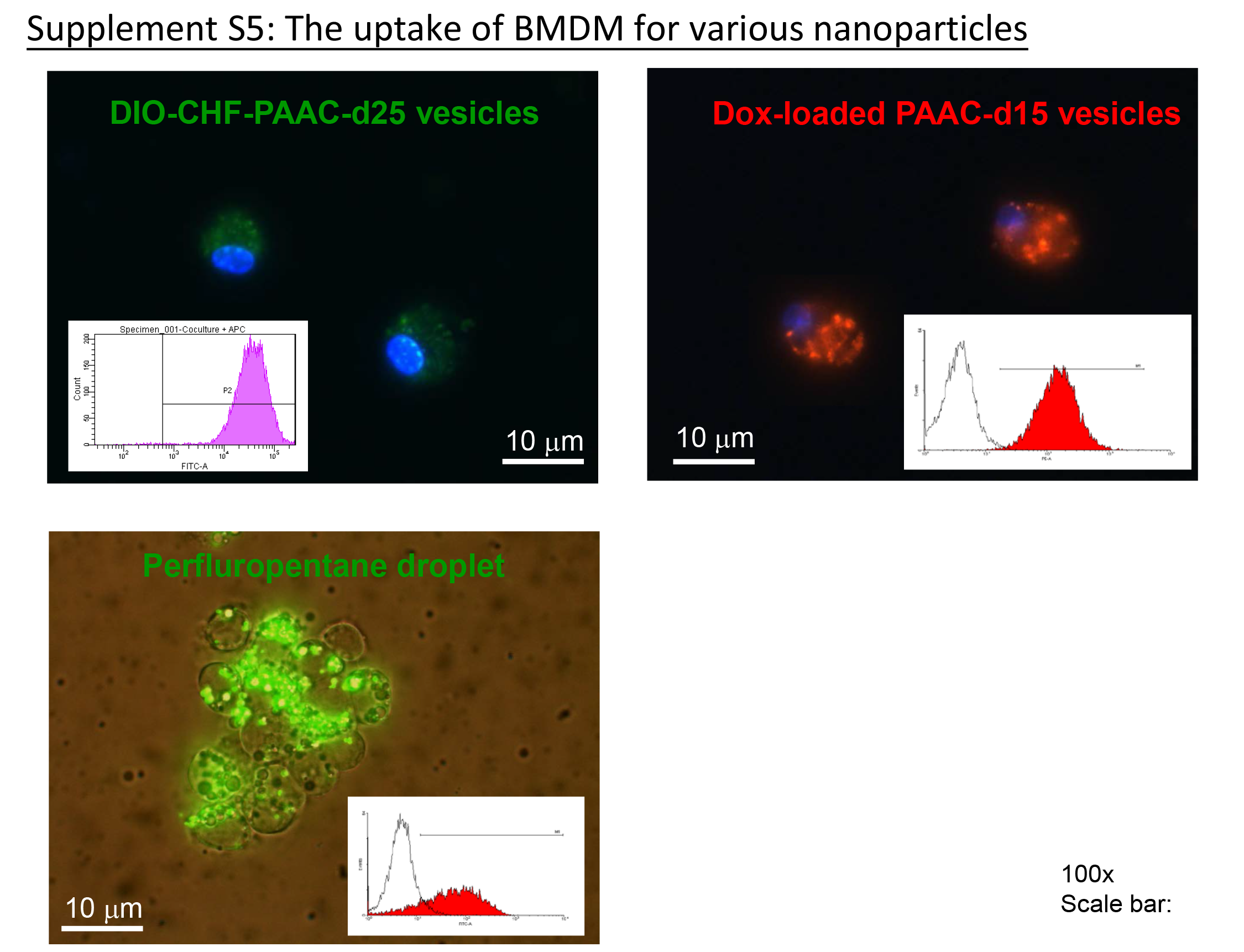

Supplement: S5 Fig — The uptake of BMDM for various nanoparticles. (A) The fluorescent microscopy of DIO-CHF-PAAC-d25 vesicles uptaken by BMDMs. The inner figure is the histogram of flow cytometry result for DIO fluorescence. (B) The fluorescent microscopy of Dox-loaded PAAC-d15 vesicles vesicles uptaken by BMDMs. The inner figure is the histogram of flow cytometry result for Dox fluorescence. (C) The fluorescent microscopy of Perfluropentance droplet uptaken by BMDMs. The inner figure is the histogram of flow cytometry result for DIO fluorescence. (TIF) [file pone.0139043.s005.tif]

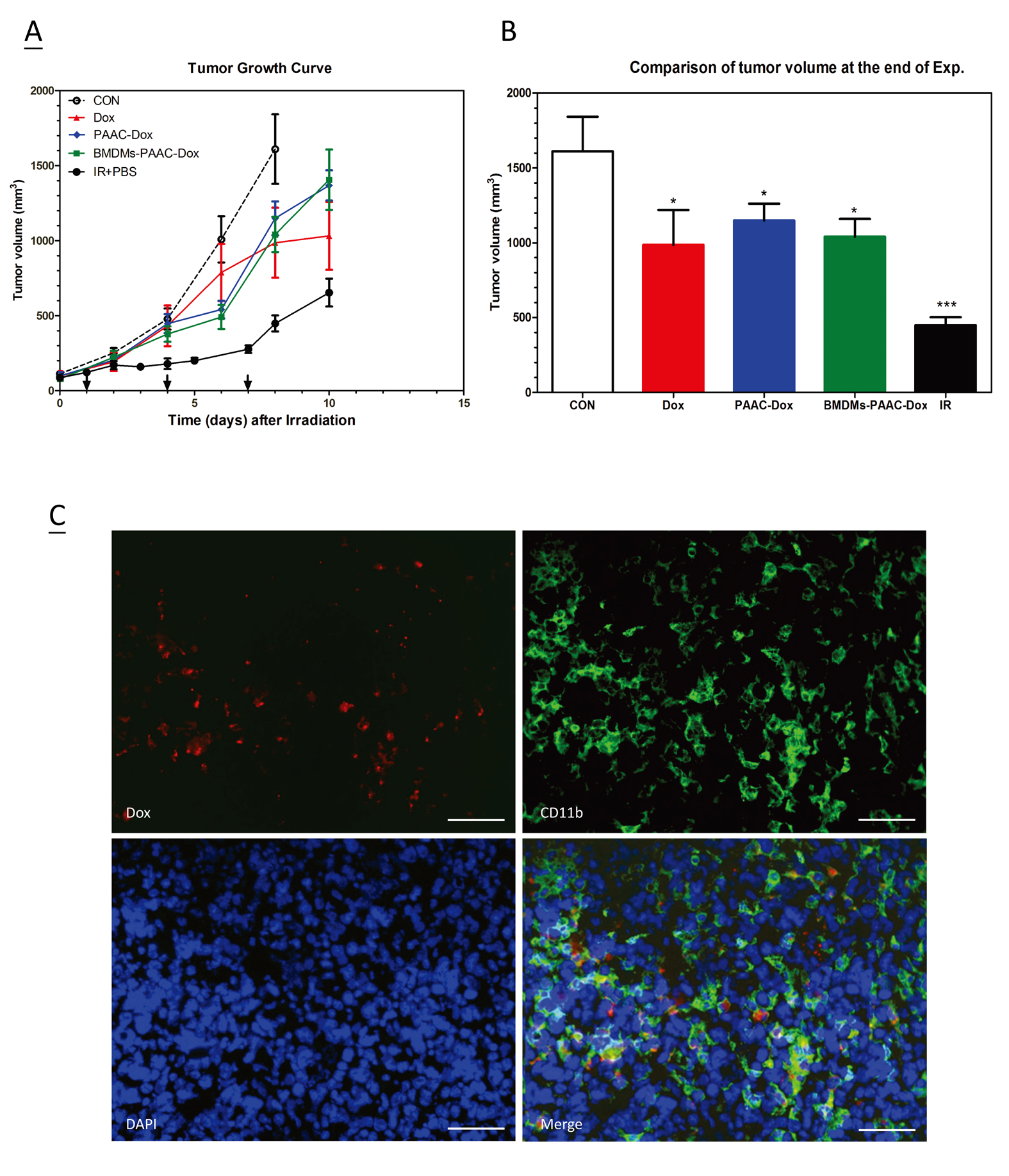

Supplement: S6 Fig — (A) Tumor growth curve of TRAMP-C1 tumors growing subcutaneously in the thigh following various single treatment. RT: 25 Gy of radiation was given when tumor diameter is around 5 mm. PBS, Dox, PAAC-Dox or BMDMs-PAAC-Dox was given intravenously at 1, 4, and 7 days after sham radiation treatment. (B) Comparison of tumor volume at the end of experiments. **: P<0.01; ***: P <0.005; n.s.: P >0.05 by one way ANOVA test. ***: P <0.005; *: P<0.05 by one way ANOVA test. (C) Representative fluorescent imaging of tumor tissues obtained from one day after the first administration of BMDMs-PAAC-Dox. scale bar = 50 um. (TIF) [file pone.0139043.s006.tif]
